# EHMTI-0309. The management of chronic headache: the current state in the provincial hospital of ouarzazate-city

**DOI:** 10.1186/1129-2377-15-S1-D26

**Published:** 2014-09-18

**Authors:** I Hajjaj

**Affiliations:** 1 Department of medicine, Sidi Hssain Benacer Hospital, Ouarzazate, Morocco

## Introduction

The province of Ouarzazate is located in the center of the Kingdom of Morocco, a population of over 350,000 inhabitants; it is considered by the Moroccan Ministry of Health as difficult area to provide health human resources.

## Aims

first study of this kind in this city; its aim is to describe the current situation of headaches among the population of Ouarzazate, to recognize the difficulties of health care and offer solutions.

## Methods

it is a retrospective study of over two years (conducted between January 2011 and December 2012), the methodology consisted of analyzing patient files in neurological consultation at Sidi Hssain Benacer hospital. Acute headaches are excluded from this study.

## Preliminary results

headache was the first reason for neurological consultation; it represented 25% of all causes of consultation. There was a female predominance of 76%. The age of the patients ranged from 3 years to 80 years, with an average age of 37. Most patients had consulted a general practitioner before seeing a neurologist or were under self-medication. Tension headache was the most frequent type of headache (42%). Comorbidity with psychiatric disorders was common. Brain CT scan was often requested by general practitioners before addressing the patient to the neurologist.

## Conclusions

To improve the global care of headache, objectives for the short, medium and long term are suggested. Achieving successful results also requires detailed studies and surveys in the region and the involvement of all stakeholders: neurologists and general practitioners, Ministry of Health, the media and civil society.

No conflict of interest.

